# Urine microRNA Profiling Displays miR-125a Dysregulation in Children with Fragile X Syndrome

**DOI:** 10.3390/cells9020289

**Published:** 2020-01-24

**Authors:** Noora Putkonen, Asta Laiho, Doug Ethell, Juha Pursiheimo, Anna-Kaisa Anttonen, Juho Pitkonen, Adriana M. Gentile, Yolanda de Diego-Otero, Maija L. Castrén

**Affiliations:** 1 Faculty of Medicine, Physiology, University of Helsinki, FI-00014 Helsinki, Finland; noora.putkonen@gmail.com (N.P.); juho.pitkonen@helsinki.fi (J.P.); 2 Turku Centre for Biotechnology, University of Turku and Åbo Akademi University, Tykistökatu 6A, FI-20520 Turku, Finland; asta.laiho@utu.fi; 3 Leucadia Therapeutics Inc., Riverside, CA 92506, USA; dougeth64@gmail.com; 4 The Joint Clinical Biochemistry Laboratory of University of Turku, University Central Hospital and Wallac Oy, FI-20520 Turku, Finland; juhpursi@utu.fi; 5 Department of Clinical Genetics, University Hospital of Helsinki, 160, FI-00290 Helsinki, Finland; anna-kaisa.anttonen@hus.fi; 6 Institute of Biomedical Research of Malaga (IBIMA) and Mental Health Unit, Regional University Hospital of Malaga, University of Malaga, Research lab. Hospital Civil, Pab-6 sot. 29009 Malaga, Spain; biogentile@gmail.com; 7 Rinnekoti Foundation, FIN-02980 Espoo, Finland; 8 Division of Biomedical Sciences, School of Medicine, University of California, Riverside, CA 92521, USA

**Keywords:** disease biomarker, urine miRNA, fragile X syndrome, autism, miR-125a

## Abstract

A triplet repeat expansion leading to transcriptional silencing of the *FMR1* gene results in fragile X syndrome (FXS), which is a common cause of inherited intellectual disability and autism. Phenotypic variation requires personalized treatment approaches and hampers clinical trials in FXS. We searched for microRNA (miRNA) biomarkers for FXS using deep sequencing of urine and identified 28 differentially regulated miRNAs when 219 reliably identified miRNAs were compared in dizygotic twin boys who shared the same environment, but one had an FXS full mutation, and the other carried a premutation allele. The largest increase was found in miR-125a in the FXS sample, and the miR-125a levels were increased in two independent sets of urine samples from a total of 19 FXS children. Urine miR-125a levels appeared to increase with age in control subjects, but varied widely in FXS subjects. Should the results be generalized, it could suggest that two FXS subgroups existed. Predicted gene targets of the differentially regulated miRNAs are involved in molecular pathways that regulate developmental processes, homeostasis, and neuronal function. Regulation of miR-125a has been associated with type I metabotropic glutamate receptor signaling (mGluR), which has been explored as a treatment target for FXS, reinforcing the possibility that urine miR-125a may provide a novel biomarker for FXS.

## 1. Introduction

MicroRNAs (miRNAs) are short (19–24 nucleotides in length) noncoding RNAs that regulate translation by promoting mRNA degradation and attenuating protein translation [1,2]. Active trafficking of miRNAs contributes to intercellular communication by local control of miRNA-mediated regulation of target genes and signaling events. Several miRNAs reach the circulation using microvesicle-dependent or RNA-binding-protein-associated active secretion or by passive leakage from cells [3]. Measurable levels of miRNAs can be isolated from bodily fluids, including urine [4]. Mounting evidence indicates that miRNAs are involved in the pathophysiology of several disorders, and disease-specific extracellular miRNA profiles have been identified [5]. Changes in circulating miRNAs have been implicated in metabolic crosstalk between organs as well as in neurodegeneration [6,7]. The accessibility and stability of circulating miRNAs support their use as biomarkers for patient stratification to improve the efficacy of targeted treatments [3].

Fragile X syndrome (FXS) is the most common cause of inherited intellectual disability and the best-known single-gene cause of autism spectrum disorder, with a prevalence of 1/4000 males and ~1/6000 females. The behavioral phenotype of FXS includes hyperactivity, attention disorders, social anxiety and mood instability, and abnormalities in sensory stimuli [8,9]. The severity of these features, as well as responses to current pharmacological treatments, varies individually. Epilepsy associates with FXS in about 15%–20% of FXS males [10]. In most cases, expansion of a triplet CGG repeat in the untranslated region of the *FMR1* gene from the normal 5–55 repeat range to >200 leads to transcriptional silencing and lack of FMR1 protein (FMRP), which results in FXS [11]. A CGG repeat between 55 and 200 triplets is a fragile X premutation that can be inherited from the mother as an expanded full mutation [12]. Premutation carriers do not show early-childhood-onset intellectual disability syndrome, and paradoxically, they have abnormally high levels of FMR1 mRNA, which predisposes them to a late-onset neurodegenerative disorder called fragile X-associated tremor/ataxia syndrome (FXTAS) [13].

FMRP expression is widespread with high abundant expression in neurons and testes [14]. The absence of functional FMRP impairs normal synaptic formation and plasticity in the central nervous system (CNS) and results in macro-orchidism due to the overproduction of Sertoli cells in testes [9,15]. FMRP is a messenger RNA (mRNA)-binding protein and controls protein translation by interacting with specific mRNAs. FMRP also associates with miRNAs and proteins incorporated into a multiprotein complex called an RNA-induced silencing complex (RISC). There is evidence that disruption of miRNA pathways contributes to the abnormal synaptogenesis in FXS [16,17,18]. A RISC consisting of miR-125a and FMRP on postsynaptic density-95 (PSD-95) mRNA has been identified as a selective mechanism that controls PSD-95 mRNA translation for signaling of group 1 metabotropic glutamate receptors (mGluR) at synapses [19]. Expression of miR-125a is decreased in synaptoneurosomes in the brain of *Fmr1* knockout (KO) mice, indicating that dysregulation of miR-125a is involved in impaired synapse function in FXS. DeMarco et al. showed very recently that phosphorylation status of FMRP regulates the stability of the miR-125a-guided RISC and PSD-95 mRNA complex [18]. Since mGluR signaling alters FMRP phosphorylation, augmented mGluR signaling in FXS may affect turnover and/or cellular cycling [20] of miR-125a.

We compared urine miRNA profiles of a boy with FXS and his twin brother, a premutation carrier, and found 28 differentially expressed miRNAs. The most pronounced increase was found in levels of miR-125a in urine of the FXS twin as well as a larger group of FXS donors. A detailed analysis of miR-125a levels revealed an age-dependent increase in control urine, whereas the scattered distribution of FXS samples suggested that two subgroups of FXS subjects might exist. Our results implicate miR-125a dysregulation in the pathophysiology of FXS, consistent with previous studies of FXS mice, and provide evidence that urine miR-125a could be a potential novel biomarker in FXS clinical trials.

## 2. Materials and Methods

### 2.1. Human Urine Samples

The research using human urine was approved by the Ethics Committee of Helsinki and Uusimaa Hospital District and by the Ethics Committee of the Regional University Hospital of Málaga. Written informed consent was signed by parents and donors to obtain human samples. One set of urine samples was collected from males and a female with FXS (*n* = 9), a premutation carrier (*n* = 1; the twin boy), and healthy controls (*n* = 8) recruited under auspices of Rinnekoti Foundation. Another independent set of urine samples obtained from control (*n* = 8) and FXS donors (*n* = 10) was analyzed at the Regional University Hospital of Malaga (Spain) for replication purposes. The APHS Biobank has coordinated the collection, processing, management and assignment of the biological samples used in this study, according to the standard procedures established for this purpose. Morning urine was collected in preservation tubes (Norgen Biotek Corp, Thorold, ON, Canada) and stored at 4 °C until miRNA extraction.

FXS molecular diagnostics was performed on blood samples and the detection of CGG repeats in the *FMR1* gene was made using the Fragile X PCR test (Abbott Laboratories, IL, USA). The triplet repeat primed PCR assay provided accurate sizing of CGG repeats; size accuracy on 5–70 CGG repeat alleles + 1 CGG repeat and on 71–230 repeat alleles 3 CGG repeats by capillary electrophoresis, alleles > 231 resolved by agarose gel with low accuracy for sizing, according to the kit information provided by the manufacturer.

### 2.2. RNA Extraction and Quantitative Real-Time PCR

Urine miRNAs were extracted with Urine microRNA Purification Kit (Norgen Biotek Corp., Ontario, Canada) according to the manufacturer’s instructions. Isolated miRNA samples were eluted in 40 μl of RNase-free water. RNA concentrations were measured with Nanodrop Spectrophotometer (Thermo Scientific, Wilmington, DE, USA) and equal amounts of miRNA (10 ng) were used in reverse transcription reactions. The levels of mature miRNAs were analyzed using individual TaqMan microRNA Assays (Applied Biosystems, Life Technologies Ltd., Carlsbad, CA, USA) according to the manufacturer’s instructions. Specific miRNA primers were used to reverse-transcribe 10 ng of total miRNA with the TaqMan MicroRNA Reverse Transcription Kit (Applied Biosystems, Life Technologies Ltd., Carlsbad, CA, USA) with specific primers (Table S1). The expression levels of miRNAs were analyzed by quantitative real-time PCR (RT-qPCR) in 96-well plates on Light Cycler 480 II Real-Time PCR System (Roche Diagnostics GmbH, Mannheim, Germany). The TaqMan probes (FAM) were detected with 465–510 nm. The miRNAs were normalized to mean Ct values of all studied miRNAs in a urine sample, as previously reported [21,22].

### 2.3. Deep Sequencing

Fragment libraries were prepared for each sample with TruSeq Small RNA Library Prep Kit (Illumina) and sequenced using the HiSeq 2500 system (Illumina, Inc., San Diego, CA, USA) as instructed by the manufacturer. Read lengths were 36 bases.

### 2.4. Analysis of Data

Raw sequence data were imported in CLC Genomics Workbench (version 7.0.3; https://www.qiagenbioinformatics.com/) for read quality inspection, adapter trimming, and miRNA annotation based on miRBase (http://www.mirbase.org/). The count data grouped on mature miRNAs were then exported to R/Bioconductor v. 3.1.0 (www.bioconductor.org) (R Core Team, 2015), where the normalization and statistical testing were carried out with the DESeq2 package [23]. The sequencing dataset was deposited in the GEO database (accession #GSE143347).

Differentially expressed miRNAs were filtered requiring absolute fold-change > 2 and Wald test *p-*value < 0.05. Target gene and pathway analysis was carried out using miRSystem [24] and DAVID gene annotation tool [25]. In miRNA system, queried miRNAs were first converted to the last miRBase annotation (version 17) and significantly enriched signaling pathways were identified. In addition to the hypergeometric *p*-values, empirical *p*-values of each function/pathway were determined by ranking the enriched hypergeometric probability as compared with null baseline probabilities [24]. The weight of a miRNA was determined by dividing the absolute expression value of the miRNA by the absolute sum of the expression values of all input miRNAs. Thereafter, the ranking score was obtained by summation of the weight of miRNA times its enrichment –log (*p*-value) from the predicted target genes. Linear regression analysis of urine miR-125a levels was performed using GraphPad Prism 5.0. Data are expressed as the mean + SEM and were analyzed for statistical significance using Student’s *t* test. A *p*-value < 0.05 was considered significant.

## 3. Results

### 3.1. Profiling of FXS Urine miRNAs by Deep Sequencing

**Figure 1:**
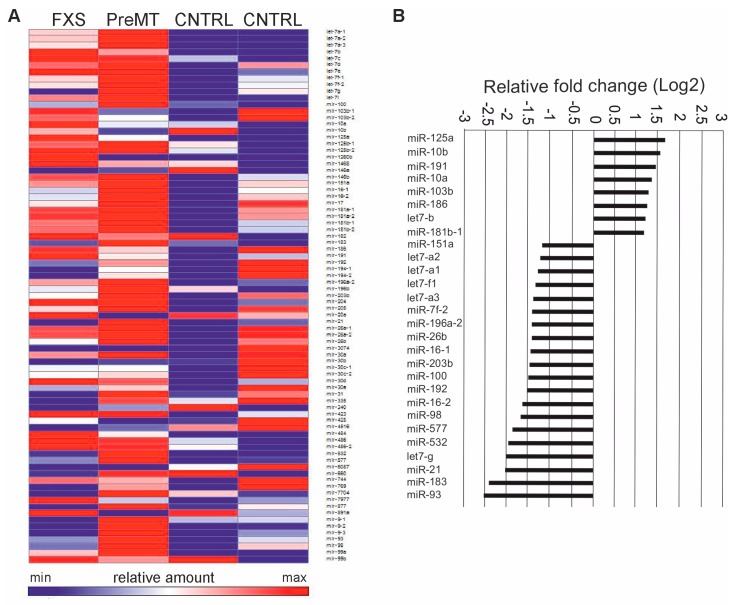
Profiling of fragile X syndrome (FXS) urine miRNAs by deep sequencing. (**A**) Comparison of identified urine miRNAs in urine of FXS full mutation (FXS) male and premutation (PreMt) carrier to controls (CNTRL). (**B**) Differentially expressed miRNAs in urine of the FXS boy compared to that of his twin brother.

To uncover FXS-specific alterations in urine miRNAs, miRNA-sequencing of urine samples obtained from individuals with and without FXS was performed by massive parallel sequencing using a HiSeq 2500 system. The initial analysis provided evidence of age-dependent clustering of FXS samples, although the sequencing read counts were highly variable across subjects. The donors included seven-year-old twin boys whose total urine miRNA reads were similar, suggesting less variation in metabolic and epigenetic measures affecting miRNA levels. One of the twin boys was diagnosed with FXS by molecular diagnostics of >200 CGG repeats (300 repeats) in the *FMR1* gene, while the other twin boy without clinical FXS diagnosis carried CGG repeats in the range of the *FMR1* gene premutation (175 repeats). The comparison of urine of the twin boys revealed 28 differentially regulated miRNAs in a total of 219 miRNAs analyzed with >50 read counts each (Figure 1A). Of those 28 notable miRNAs, 8 were upregulated and 20 were downregulated in FXS urine compared to non-FXS urine (Figure 1B). The largest increase was found in the levels of hsa-miR-125a-5p.

### 3.2. Increased miR-125a in Urine of Children with FXS

**Figure 2:**
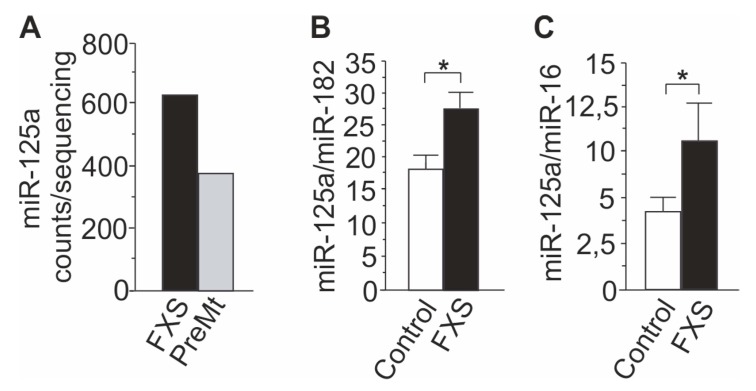
Increase in miRNA-125a levels in FXS urine. (**A**) Comparison of miR-125a levels in urine of the FXS boy vs. his twin brother by deep sequencing. (**B**) A bar graph showing the increase in the levels of miR-125a in urine of FXS subjects compared to controls normalized by miR-182 and (**C**) after normalization with miR-16. Control (*n*) = 8; FXS (*n*) = 9. Data are expressed as the means SEM. Asterisks indicate a statistically significant difference (*p* < 0.05) with Student’s *t* test.

The RT-qPCR analysis confirmed the abnormal increase in miR-125a levels in urine of the FXS twin boy found by deep sequencing (Figure 2A). The miR-125a levels were 1.6-fold higher in urine of the FXS twin boy than those of his twin brother, and an identical increase was seen in replicate samples taken one year after the first sampling. The miR-125a levels were also found to be increased in a larger number of FXS samples from donors with a homogenous Finnish genetic heritage (Table 1) compared to healthy controls (Figure 2B). The data were normalized to miR-182 that was not regulated in the sequencing analysis, and the increase was more pronounced when miR-16 was used for normalization consistent with the reduction of miR-16 in FXS urine in the sequencing analysis (Figure 2C).

The miR-125a urine analysis was replicated in an independent pool of FXS urine samples collected by a Spanish lab at the Regional University Hospital of Malaga-IBIMA. The comparison of urine samples of Spanish FXS children (*n* = 10) aged 2–7 years and healthy controls (*n* = 8) aged 2–6 years confirmed the 1.6-fold increase (*p* = 0,01) in miR-125a levels in urine: control 13.9 (SD = 3.7, SEM = 1.51) and FXS 22.5 (SD = 2.9, SEM = 1.02). The levels of miR-182 did not differ between FXS and control samples. Altogether, the data suggested that urine miR-125a levels were differentially regulated in FXS urine compared to those of healthy controls, consistent with the previous studies that have shown FMRP-dependent regulation of miR-125a.

### 3.3. Age-Dependent Dysregulation of miR-125a in Urine of Children with FXS

**Figure 3:**
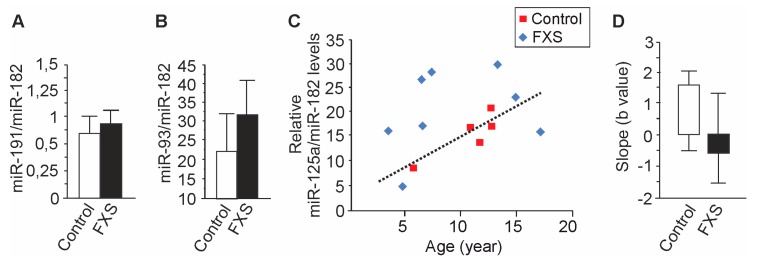
Distribution of miRNA levels. (**A**) Levels of miR-191 and (**B**) miR-93 did not statistically differ between control and FXS urine. Control (*n*) = 8; FXS (*n*) = 9. (**C**) Age-dependent relative levels of miR-125a in urine of healthy donors (Control) and FXS cases. (**D**) The variation of the slope of the lines (95 confident band). Data are expressed as means ± SEM.

The relative levels of miR-191 and miR-93 did not differ between FXS and control urine in the expanded pool of samples (Figure 3A), suggesting that the miRNA differences identified in urine of twins were small and that miRNA levels may show high variability. The difference of urine miR-125a levels between FXS and control urine was confirmed in two sets of samples, increasing the reliability of the results, and larger studies are needed to explore the impact of the other miRNAs identified to be dysregulated individually. Furthermore, since urine miRNAs of premutation carrier and FXS twin boys were initially compared, the assessment of the role of each miRNA has to be done in a set of samples which includes both premutation carriers and FXS individuals.

The distribution of miR-125a in urine of healthy children aged 4–18 years (*n* = 5) showed an age-dependent linear increase (1.559*X – 2.147; R^2^ = 0.779; *p* = 0.0473) whereas the miR-125 levels in FXS samples were scattered (Y = −0.648*X + 29.18; R^2^ = 0.074; *p* = 0.478), and the samples did not correlate (Pearson’s correlation r = −0.209) (Figure 3B). Urine miR-125a levels of two FXS boys were lower than the levels of controls and other 5 FXS donors, while all other FXS donors had higher urine miR-125a levels than the age-matched controls. The small sample size in the study associated with a high variation of the slope of the line in the analysis of FXS samples, leading to significant limitations in the interpretation of the results. However, the distribution of urine miR-125a samples indicated a possibility that the FXS samples could be divided into two groups, but larger studies are needed to investigate and possibly confirm the potential of miR-125a as a tool for stratification of FXS males.

In our set of samples, low levels of urine miR-125a did not associate with genetic mosaicism of the FMR1 full mutation, and there was no correlation between urine miR-125a levels and specific pharmacological intervention in the sample pool (Table 1). Levels of miR-125a were low in urine of a 17-year-old FXS boy with no medications as well as in urine of a 5-year-old FXS boy treated with risperidone for aggressive behavior combined with methylphenidate for hyperactivity. On the other hand, urine miR-125a levels were abnormally increased in two FXS donors with methylphenidate treatment for hyperactivity. The controls did not have any medications. Only one FXS boy (FXS6) had epilepsy, and his miR-125a levels were in the range of the high FXS values.

### 3.4. Target Gene and Pathway Analysis of miRNAs Regulated Differentially in FXS Urine

**Figure 4:**
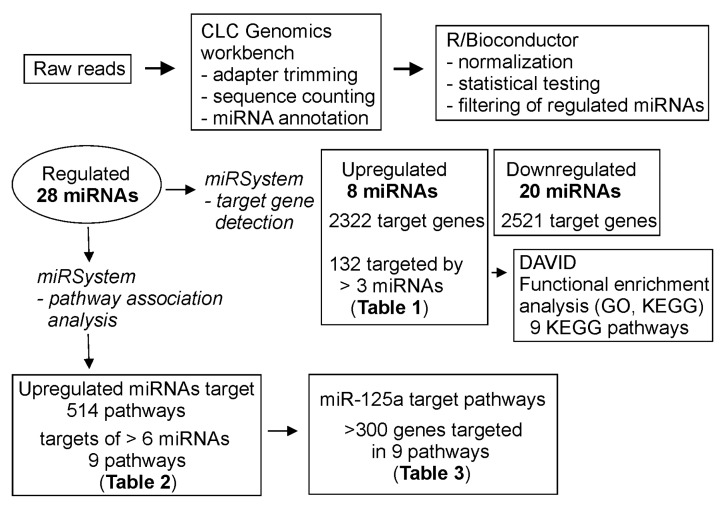
Schematic presentation of the analysis of urine sequencing data.

We analyzed predicted target genes and pathways associated with the miRNAs that were differentially regulated in urine of the FXS twin boys using miRSystem and DAVID gene annotation tool as shown in the summary of the sequencing data analysis in Figure 4 (http://mirsystem.cgm.ntu.edu miRNAs) [24,25].

Target gene analysis of increased miRNAs (all but miR-103b-1) identified 2322 possible targets. Genes targeted by five miRNAs included *gamma-aminobutyric acid* (*GABA*) *A receptor, beta 2* (*GABRB2*) and *StAR-related lipid transfer* (*START*) *domain containing 13* (*STARD13*), and genes targeted by 4 miRNAs included *BCL2L2*, *BDNF*, *GRIN3A*, among others (Table 2). *Metabotropic glutamate receptor 3* (*GRM3*) was targeted by two miRNAs.

We chose 132 genes targeted by three or more miRNAs that were increased in FXS urine for initial pathway analysis using DAVID gene annotation tool24, GO term, and KEGG pathway annotations [26]. We found nine KEGG pathway terms enriched with these genes: MAPK signaling pathway, pathways in cancer, Wnt signaling pathway, focal adhesion, glioma, chronic myeloid leukemia, neurotrophic signaling pathway, pancreatic cancer, and TGF-beta signaling pathway. The miRsystem tool found 514 pathways where the miRNAs were involved. A functional annotation summary of pathways of six enriched miRNAs is shown in Table 3.

The pathway ranking summary revealed that all six miRNAs were involved in nine pathways: signaling by insulin receptor, focal adhesion, MAPK signaling pathway, neurotrophin signaling pathway, signaling to ERKs, pathways in cancer, signaling by NGF, NGF signaling via TrkA from the plasma membrane, and hemostasis (Table 4).

Pathway analysis of miR-125a showed similarities to the predicted pathways of the group of increased miRNAs, including pathways of developmental biology, adaptive immune system, hemostasis, transmembrane transport of small molecules, GPCR ligand binding, cell cycle mitotic, cancer, neuroactive ligand-receptor activation, and class A1 of rhodopsin-like receptors (Table 5). Furthermore, the predicted pathways for increased and reduced miRNAs showed similarities. MAPK signaling pathway and pathways in cancer and developmental biology had the highest scores in the pathway ranking summary for the reduced miRNAs.

## 4. Discussion

The present study identified FXS-specific changes in urine miR-125a. The levels of miR-125a were found to be abnormally increased in FXS urine when urine samples of twin boys and an expanded pool of donors collected in two independent labs were compared. Previous studies have demonstrated that regulation of miR-125a is affected in the absence of FMRP, which stressed the potential importance of urine dysregulation of miR-125a among 28 differentially regulated miRNAs identified by deep sequencing. Levels of miR-125a are shown to be reduced in synaptoneurosomes isolated from the FXS mouse brain [19], and the phosphorylation state of FMRP regulates the stability of miR-125a-guided RISC-PSD 95 mRNA complex, which is critical for synapse function [18]. Increased urine miR-125a levels may reflect increased production and/or secretion of miR-125a, but it is not possible to make any direct correlations between human urine and mouse brain miRNA levels. Furthermore, a detailed analysis of miR-125a levels in children’s urine revealed an age-dependent regulation, whereas FXS samples did not show linear correlation indicating higher individual variability. Urine miR-125a levels of two FXS males were lower than those of the healthy controls and of five other FXS males. There was no clear correlation between the miR-125a levels and the length of the repeat expansion in the *FMR1* gene, genetic mosaicism, or pharmacological interventions within the small set of samples. The FXS children with low levels of miR-125a in urine may present a subgroup whose cellular homeostasis differs from that of the subgroup with higher levels of miR-125a. A well-described association between miR-125a- and mGluR5 signaling [19] suggests that urine miR-125a levels may provide a novel tool to subgroup FXS children based on individual differences linked particularly to mGluR5 signaling, which is considered to be the most critically dysregulated signaling pathway in FXS.

Expression of miR-125a is high in the ovary, epididymis, spleen, and in some endocrine organs and regions of the brain (www.microRNA.org). There is evidence that members of the miR-125 family can have disease-suppressing properties, implying that it could predict disease onset or have prognostic value during disease progression [27,28]. Recently ectopic expression of miR-125a was found to promote granulocyte differentiation, and improved understanding of miR-125a function may assist in the development of novel miR-125a-targeted therapies [29]. A role for miR-125a-5p has been identified in the regulation of endothelial tightness, supporting the potential of miR-125a as a disease biomarker in circulating biofluids [28,30]. There is evidence that miR-125a reduces endothelin-1 expression and immune cell efflux in inflammation. It has been shown that miR-125a regulates the secretion of some inflammatory cytokines (interleukin (IL)2, IL6, TNF-alpha, and TNF-beta) [31]. In mouse in vivo and in vitro models of thyroiditis, an increased miR-125a expression reduces autophagy and cell proliferation and increases the apoptotic rate and the expression of proinflammatory factors tumor necrosis factor-α, IL-1β, IL-6, and IL-18 via downregulation of the phosphoinositide 3-kinase/protein kinase B/mammalian target of rapamycin (PI3K/Akt/mTOR)signaling pathway [32]. Low levels of miR-125a-5p are found in different types of tumors [33]. Levels of miR-125a-5p are also found to be decreased in the hippocampus of rats with pentylenetetrazol (PTZ)-induced epilepsy, whereas miR-125a-5p overexpression can attenuate seizures and decrease inflammatory factors in these rats [34].

In the present study, a total of 219 miRNAs with the expression of more than 50 read counts were identified in human urine by RNA seq. At least a 1.5-fold expression difference cutoff is suggested by several miRNA profiling studies that have explored the impact of changes of miRNA levels on cellular biology [35]. We observed 28 differentially expressed miRNAs in urine of an FXS boy compared to urine of his twin brother. The most increased miRNA in FXS urine was miR-125a, and its increase was confirmed by RT-qPCR. The smaller increase of miR-191 levels and reduction of miR-93 levels in FXS urine were not confirmed in the expanded pool of samples, suggesting that urine miRNA levels are low and show high individual variability affecting their analysis. Therefore, dysregulation of the miRNAs other than miR-125a remains to be investigated in a larger set of samples which include both premutation carriers and FXS individuals. A panel of circulating miRNAs may have more potential to show efficacy than single miRNAs in drug response monitoring as observed in biomarker studies of cancer patients [19]. Many predicted target genes of the differentially regulated miRNAs were shown to be involved in pathways that regulate molecular and cellular processes known to be disrupted in FXS, including axon guidance and neurotrophin signaling in the nervous systems. The data suggest that urine miRNA levels may reflect common pathological miRNA-dependent processes caused by the absence of FMRP in multiple tissues [36].

Although most miRNAs are intracellular, significant levels of miRNAs appear outside cells and circulate in human body fluids, including urine [4,20,37]. Each body fluid has its own miRNA composition, but the origin of circulating miRNAs is not well understood, although correlations between circulating and tissue miRNAs exist [38]. There is evidence that miRNAs can reach the circulation from active secretion or passive leakage from broken cells [3]. Certain miRNAs are targeted for export and actively secreted to extracellular fluids [39]. Active miRNA secretion can be mediated via microvesicles or a microvesicle-free, RNA-binding protein-dependent pathway. The majority of urinary miRNAs originate from renal and urethral cells, but other tissues can also actively release circulating extracellular miRNAs packaged in exosomes (lipid vesicles) [40] or bound to RNA-binding proteins [35,41] into urine via renal epithelial cells. Interestingly, the profiles of miRNAs in urine and cerebrospinal fluid show many similarities, such as low miRNA abundance [4]. Both cell-free and exosomal preparations are found in urine samples [3], and the small number of miRNAs may indicate that only distinct miRNAs are stabilized by microvesicles or associated with RNA-binding protein and high-density lipoprotein (HDL) as a carrier and protected from degradation by ribonucleases in the circulation.

The present study did not examine correlations of miR-125a levels in urine and other bodily fluids. Previously slight but not significant reduction of miR-125a-5p was observed in FXTAS patients’ blood by deep sequencing [42]. The miRNA profiling of serum in children with autism spectrum disorder (ASD) identified thirteen differentially expressed miRNAs in individuals with ASD compared to the controls, and miR-125a was not among the dysregulated miRNAs [43].

There are 12 brain miRNAs identified to interact with FMRP [16], and these miRNAs include miR-125a. Involvement of miR-125a in fate determination of neuronal lineages [44] and synaptic plasticity [45] links dysregulation of miR-125a to FXS but potentially also to several other neurodevelopmental disorders. Differential expression of miR-125a in the male and female frontal lobe region during normal development has been reported [46], and many similar sexually dimorphic miRNAs are associated with autism-related diseases and processes [47]. Gender effects on the analysis of FXS urine miR-125a levels were reduced in the current study by using only male controls. Only one female FXS subject was included in the study, and interestingly, her urine miR-125a levels were just slightly above the age-matched control levels. 

## 5. Conclusions

Our study is the first to examine and show disease-specific changes in the urinary miRNA profile in a neurodevelopmental disorder. Levels of miR-125a were shown to be increased in FXS urine, consistent with previous observations showing the involvement of FMRP in the regulation of miR-125a expression. Altered peripheral miRNA levels have been detected in several neuropsychiatric disorders, including depression, schizophrenia, and ASD [48,49], and increased plasma miR-125a levels were very recently shown in patients with bipolar disorder and particularly in bipolar manic patients [50]. The results of the present study demonstrate the potential of urine miRNA profiling for miRNA biomarker development in FXS. Larger studies are necessary to confirm the abnormalities of urine miR-125a levels and to explore the impact of the other dysregulated miRNAs observed in urine profiling in FXS.

## Figures and Tables

**Table 1:** Summary of the *FMR1* mutations and medications of the nine Finnish FXS donors.

Subject/phenotype	Age; Years	Gender	Repeat Length	Medication
FXS1	4	male	mosaic	
FXS2	5	male	>200	risperidone, methyphenidate
FXS3	6	male	86/520	methylphenidate
FXS4	6	male	>200	
FXS5	8	male	>200	
FXS6	8	male	300	oxcarbatzepine
FXS7	13	male	>200	methylphenidate
FXS8	14	female	34/500	
FXS9	17	male	300	

**Table 2:** Target gene analysis of increased miRNAs.

Target Gene	Gene Description	Observed Number of miRNA
STARD13	StAR-related lipid transfer (START) domain containing 13	5
GABRB2	gamma-aminobutyric acid (GABA) A receptor, beta 2	5
BDNF	brain-derived neurotrophic factor	4
CECR6	cat eye syndrome chromosome region, candidate 6	4
CNOT6	CCR4-NOT transcription complex, subunit 6	4
CRK	v-crk avian sarcoma virus CT10 oncogene homolog	4
DPF2	D4, zinc and double PHD fingers family 2	4
DVL3	dishevelled segment polarity protein 3	4
ELOVL6	ELOVL fatty acid elongase 6	4
EPHA4	EPH receptor A4	4
FIGN	fidgetin	4
BCL2L2	BCL2-like 2	4
FOXP1	forkhead box P1	4
GRIN3A	glutamate receptor, ionotropic, N-methyl-D-aspartate 3A	4
IGF2BP2	insulin-like growth factor 2 mRNA binding protein 2	4
IL1RAPL1	interleukin 1 receptor accessory protein-like 1	4
KCNA1	potassium voltage-gated channel, shaker-related subfamily, member 1 (episodic ataxia with myokymia)	4
MTF1	metal-regulatory transcription factor 1	4
MYCBP	MYC binding protein	4
ONECUT2	one cut homeobox 2	4
RASL10B	RAS-like, family 10, member B	4
SORT1	sortilin 1	4

**Table 3:** Functional annotation summary (miRSystem) of pathways for 6 upregulated miRNAs in FXS urine.

Database	Pathway	Target genes	Score
REACTOME	Developmental Biology	105	3.663
REACTOME	Axon guidance	72	3.588
KEGG	MAPK signaling pathway	67	3.212
REACTOME	L1CAM interactions	32	3.050
KEGG	Pathways in cancer	73	2.827
BIOCARTA	Biocarta MAPK pathways	31	2.802
REACTOME	Interaction between L1 and ankyrins	13	2.585
REACTOME	Fatty acid triacylglycerol and ketone body metabolism	22	2.563
KEGG	WNT signaling pathway	36	2.559
REACTOME	Neuronal system	59	2.365
PATHWAY INTERACTION DATABASE	Neurotrophic factor-mediated Trk receptor signaling	16	2.331
KEGG	Focal adhesion	48	2.328
KEGG	Glioma	20	2.323
KEGG	Chronic myeloid leukemia	20	2.307
KEGG	Neurotrophin signaling pathway	29	2.241
PATHWAY INTERACTION DATABASE	C-myb transcription factor network	26	2.237
REACTOME	Signaling by NGF	49	2.194
KEGG	Pancreatic cancer	21	2.138
KEGG	TGF-beta signaling pathway	28	2.129
PATHWAY INTERACTION DATABASE	Signaling events regulated by Ret tyrosine kinase	11	2.103
REACTOME	NGF signaling via TrkA from Plasma membrane	37	2.057

**Table 4:** Pathway ranking summary (miRSystem) of pathways targeted by upregulated miRNAs identified in FXS urine.

Database	Pathway	Genes	Raw	Empirical
REACTOME	Signaling by insulin receptor	4	1.41372e-2	5.07712e-2
KEGG	Focal adhesion	6	7.34030e-3	3.22732e-2
KEGG	MAPK signaling pathway	7	8.68277e-3	8.62286e-2
KEGG	Neurotrophin signaling pathway	6	2.23557e-2	1.32203e-1
REACTOME	Signaling to ERKs	3	3.57835e-3	3.10591e-2
KEGG	Pathways in cancer	5	9.69353e-2	3.33703e-1
REACTOME	Signaling by NGF	6	1.14105e-2	5.61302e-2
REACTOME	NGF signaling via TrkA from plasma membrane	5	6.46094e-3	1.29899e-2
REACTOME	Hemostasis	5	1.66621e-1	3.67254e-1

**Table 5:** Pathway analysis (miRSystem) of miR-125a.

Database	Pathway	Genes	Raw	Empirical
REACTOME	Developmental Biology	494	8.78e-4	3.89749e-1
REACTOME	Adaptive immune system	482	2.519e-2	2.90419e-1
REACTOME	Hemostasis	467	6.955e-3	3.12984e-1
REACTOME	Transmembrane transport of small molecules	427	1.07902e-1	5.3854e-1
REACTOME	GPCR ligand binding	410	1.5103e-2	5.7303e-2
REACTOME	Cell cycle mitotic	330	1.06856e-1	4.15975e-1
KEGG	Pathways in cancer	325	3.1018e-2	5.8577e-1
KEGG	Neuroactive ligand receptor activation	318	9.2561e-2	2.12222e-1
REACTOME	Class A1 (Rhodopsin-Like receptors)	305	6.7227e-2	2.74648e-1

## Supplementary Materials

The following are available online at https://www.mdpi.com/2073-4409/9/2/289/s1, Table S1 showing primers (Life Technologies Ltd) used in the RT-qPCR.
