# Supplementary figures and images for: Urine microRNA Profiling Displays miR-125a Dysregulation in Children with Fragile X Syndrome
Source: Cells. 2020 Jan 24;9(2):289. doi: 10.3390/cells9020289 (PMC7072127; doi:10.3390/cells9020289)

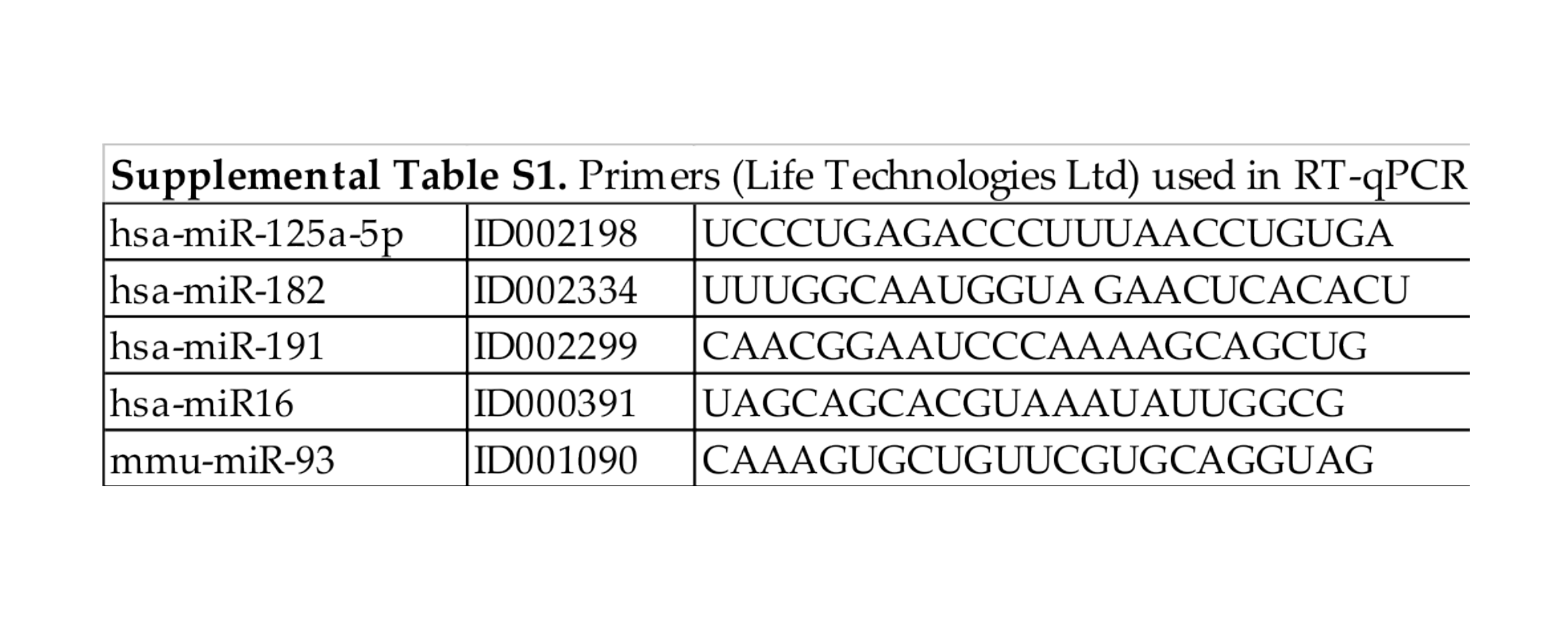

Supplement: Supplementary file 1 [file cells-09-00289-s001.zip › cells-647857-supplementary.tif]
